# Hyperthermic Effect of Ginger (*Zingiber officinale*) Extract-Containing Beverage on Peripheral Skin Surface Temperature in Women

**DOI:** 10.1155/2018/3207623

**Published:** 2018-10-08

**Authors:** Keiichiro Sugimoto, Hiroaki Takeuchi, Kazuya Nakagawa, Yasuhiro Matsuoka

**Affiliations:** ^1^Research and Development Center, Nagaoka Co. Ltd., 1-3-30, Itsukaichi, Ibaraki, Osaka 567-0005, Japan; ^2^Department of Food and Health Sciences, Jissen Women's University, 4-1-1 Osakaue, Hino, Tokyo 191-8510, Japan

## Abstract

Ginger is known to warm the body. Therefore, we conducted a placebo-controlled crossover trial to investigate the hyperthermic effect of a palatable ginger-containing beverage in healthy women with cold-sensitive extremities. Six women drank 280 mL of 0.07% ginger extract-containing or placebo beverage in a temperature-controlled room (21°C). Their palm temperatures were measured as measure of surface body temperature using a thermographic camera before intake and every 10 min after intake for 60 min. Palm temperature increased immediately following intake of the ginger and placebo beverages. However, palm temperature following intake of the ginger beverage increased for 20 min, while palm temperature following placebo intake decreased again after 10 min. The increased palm temperature following ginger intake was maintained significantly longer than after placebo intake (*p *< 0.05). In response to a questionnaire, some subjects answered that their increased body temperature was maintained after drinking the ginger beverage. Ginger extract-containing beverage may thus improve cold sensitivity.

## 1. Introduction

Cold sensitivity of the extremities (*hie-sho*) is defined as being aware of an abnormally cold sensation on specific parts of body, even in an ambient temperature in which other parts of the body do not feel cold [1]. This cold sensitivity is common in Japanese women. According to surveys, over 50% of adult women complained of coldness of their peripheral extremities and/or around their hips in winter or in an air-conditioned room [2, 3]. This condition is associated with an autonomic nervous system imbalance due to female hormonal disorders, such as menopause, delivery, menstruation, or infertility [4–6], menstrual anemia, diet restriction for weight reduction, or diet disorders [6]. Autonomic nervous system imbalance induces contraction of the peripheral vessels, leading to insufficient blood flow and heat transfer to the extremities [3, 7]. With regard to environmental factors, wearing light clothing and/or consuming cold drinks in an air-conditioned atmosphere, either at home or in the office, are also possible reasons for* hie-sho* [6, 8].* Hie-sho* has also been reported in males [9]. Severe* hie-sho* can affect the sufferer's quality of life and may cause other symptoms, such as shoulder stiffness, lumbar pain, headache, sleeplessness, or constipation [2, 6, 8]. Improving the peripheral circulation and enhancing thermogenesis are considered as effective means of alleviating cold sensitivity.

Ginger (*Zingiber officinale*) is an annual plant originating from tropical Asia. The rhizome, generally referred to as ginger, is widely used as a spice or folk medicine. In traditional Chinese medicine (Shennong Ben Cao Jing), dried ginger is used to warm the body. Ginger soup, comprising hot water with dried ginger powder or grated raw ginger, is drunk to keep warm, especially when a person has a cold. Ginger root contains pungent polyphenols known as gingerols, such as 6-shogaol, 6-gingerol, and zingeron [10]. These components have been reported to have thermogenic effects by inducing adrenaline secretion via activation of transient-receptor potential vanilloid 1 channels [11]. Human trials have also reported elevation of body temperatures using ginger-containing capsules [12–14], bread [15], or tea [16]. However, ginger has a pungent flavor and tastes unpleasant when used in high doses.

Therefore, we developed a beverage containing ginger extract with an effective dose and acceptable taste. We investigated the hyperthermic effect of this beverage on skin temperature in the limbs in healthy Japanese women with cold sensitivity.

## 2. Materials and Methods

### 2.1. Human Subjects

Eight healthy Japanese female volunteers with* hie-sho* (age: 30.0 ± 6.1 years; height: 161.0 ± 4.2 cm; weight: 53.0 ± 5.4 kg, mean ± standard deviation [SD]) were recruited from a consumer monitor database by IPSOS Nihon-Toukeichousa Co., Ltd. (Tokyo, Japan) according to the criteria described below. The selection criteria were women who were healthy, but who experienced self-reported cold sensitivity, based on a questionnaire, as reported by Nagashima et al. [17], with slight modifications. However, none of the participants had a pathological diagnosis according to the diagnostic criteria of Terasawa [2]. Participants also fulfilled the following criteria: not receiving medical treatment, not menstruating, not allergic to ginger, not taking health supplements, and a body mass index > 8.5 and < 25.0 (kg/m^2^).

The study was carried out in accordance with the Helsinki declaration, and all subjects provided written informed consent.

### 2.2. Preparation of Ginger-Containing Beverages and Sensory Evaluation

The ginger extract was prepared from ginger roots that were harvested in Vietnam, dried in hot air, and mixed with approximately 40% ethanol (w/w) at 65°C, followed by mixing with high-fructose corn syrups. The mixture was filtered through filter paper and evaporated to 75 degrees Brix. To determine a palatable dose of the ginger extract, three levels of ginger extract were blended to a base beverage (40 kcal/100 mL) composed of 13% of high-fructose corn syrup (Brix. 75), 0.07% of citric acid, 0.06% of ascorbic acid, and 0.06% of caramel color.

In a preliminary study, a beverage containing 0.116% of ginger extract was the lowest concentration that had a slightly pungent taste. A beverage that contained 0.35% of ginger extract was three times that of the lowest concentration. The highest concentration of ginger used was 0.817% extract, which was reported by Natsuno and Hirayanagi [14] as the lowest total dosage of 6-gingerol and 6-shogaol (4.76 mg/serving) among previously reported trials. A volume of 280 mL of the beverage was packed in plastic bottles and sterilized by heating at 80°C for 20 min. The three different levels of ginger content beverages were evaluated for palatability by 23 sensory panelists (22–65 years, mean age: 43.3 ± 11.7 years, 17 men and 6 women).

Evaluation was carried out between 9:00 am and 12:30 am before lunch time in a sensory evaluation room in which the environmental condition was the same as that of human trials. The beverages were served to panelists at 55°C and evaluated. The three samples were coded by the alphabet and the serving order was randomly allocated. A seven-point hedonic scale test was carried out (test scales: −3 = strongly dislike, −2 = dislike, −1 = slightly dislike, 0 = neither like nor dislike, 1 = somewhat like, 2 = like, 3 = strongly like) on drinking ease, aftertaste, pungency, and overall judgement. Analytical evaluation was also performed by rating scale methods (test scales: −3 = extremely weak, −2 = weak, −1 = somewhat weak, 0 = moderate, 1 = somewhat strong, 2 = strong, 3 = extremely strong) for aftertaste, pungency, smell, sweetness, and sourness. Panelists wrote their comments on a free description column.

### 2.3. Quantification of 6-Gingerol and 6-Shogaol (Gingerols) in the Beverage

**Figure 1:**
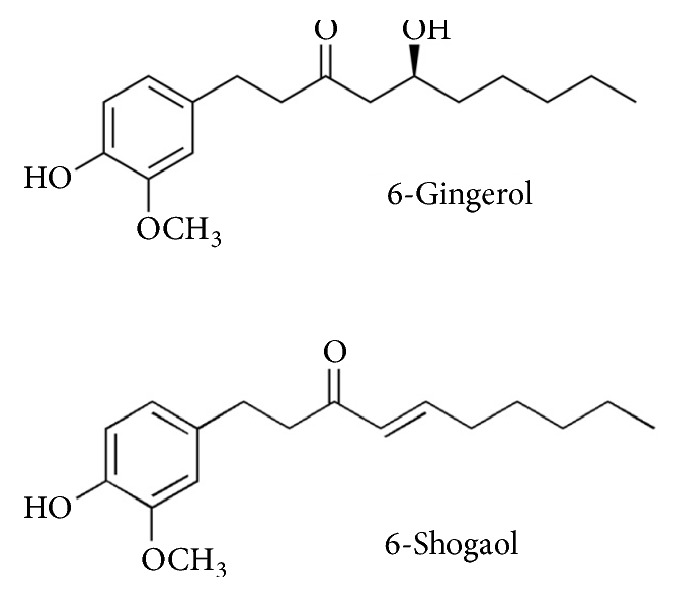
Chemical structures of 6-gingerol and 6-shogaol.

Standards for 6-gingerol and 6-shogaol (Figure 1) were purchased from Wako Pure Chemical Industries Ltd. (Osaka, Japan). A volume of 50 mL of 0.07% ginger-containing beverage was loaded onto a Sep-pack Plus C18 Short Cartridge® (Waters Co., Milford, MA, USA) conditioned with methanol followed by water. After washing with water, the absorbed component was eluted with 2 mL of 100% methanol. The 25-fold concentrated elution was filtered through a 0.45 *μ*m membrane syringe filter (Millex®-LH 0.45 *μ*m Philic PTFE 13 mm; Merck, Darmstadt, Germany) and the filtrate was recovered for high-performance liquid chromatography.

The contents of 6-gingerol and 6-shogaol were determined by reverse-phase high-performance liquid chromatography on an STR ODS-II column (150 mm × 4.6 mm internal diameter; Shinwa Chemical Industries Ltd., Kyoto, Japan) using an LC-10A system (Shimadzu Co., Ltd, Kyoto, Japan) at an absorbance of 282 nm with a UV/visible detector SPD-10A (Shimadzu). The conditions were modified slightly, as reported previously [18, 19]. The mobile phase was aqueous methanol and the gradient conditions were as follows: 0–5 min, 30%–60% methanol; 5–10 min, 60% methanol; 10–13 min, 60%–100% methanol; and 13–18 min, 100% methanol. The column temperature was 50°C. The flow rate was 1.2 mL/min, which was increased to 1.5 mL/min at 15 min. The sample injection volume was 10 *μ*L. The chromatogram was recorded using a Chromatopac C-R7A integrator (Shimadzu).

### 2.4. Protocol for the Human Trial

**Figure 2:**
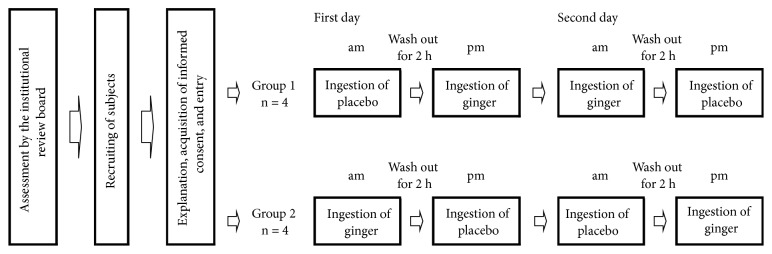
Study schedule.

**Figure 3:**
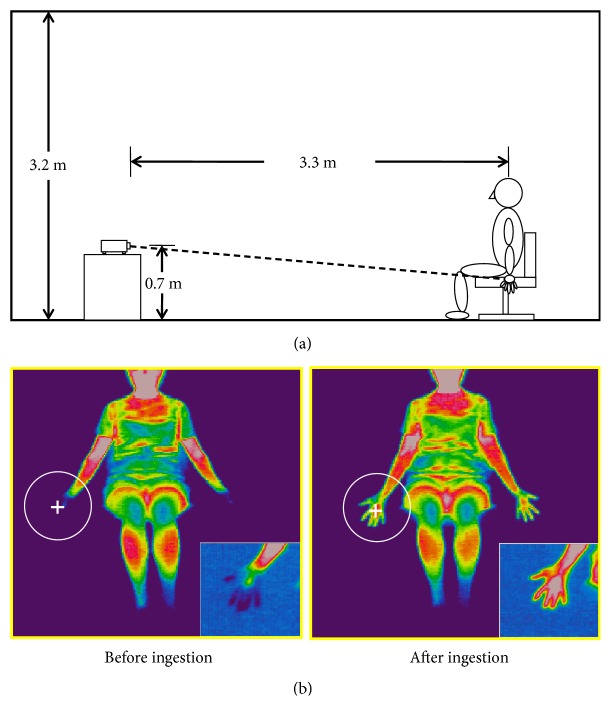
Conditions of the test room (a). Measurement site of the thermographic examination (b).

We conducted a placebo-controlled crossover trial over 2 days. The base beverage described in Section 2.2 was used as the placebo beverage and the base drink that contained 0.116% of ginger extract was used as the ginger beverage for the trials. As shown in Figure 2, subjects were randomly divided into two groups. On the first day, group 1 drank the ginger beverage in the morning, followed by the placebo beverage in the afternoon, while group 2 drank the placebo in the morning followed by the ginger beverage in the afternoon. On the second day, the subjects in each group drank the beverages in the reverse order. The subjects' tympanic temperatures were measured during the acclimatization period before the trial using a Ken-on Kun MC-510 thermometer (Omron Healthcare Co., Ltd., Kyoto, Japan). The crossover trial was carried out in an air-conditioned room (temperature, 21.0 ± 1.0°C; humidity of 50%  ± 10%) with carpet (Figure 3(a)) at 10:00 and 14:00 h local time, to take into account diurnal variation. Subjects wore T-shirts and shorts, had bare feet, and were isolated from any other furniture in the test room between the morning and afternoon trials. The temperature in the middle of the subject's palm (Figure 3(b)) was measured using a thermotracer TH-5100 (NEC San-ei Co., Tokyo, Japan) at a distance of 3.3 m. Images were analyzed using the image-processing software TH-5100 (NEC San-ei Co.).

Subjects entered the test room 1 h before the trial and acclimated while sitting, under resting conditions. Both beverages were served as hot drinks at 55°C and were covered with a holder to avoid exposing the subject's hand to the heat. The beverages were consumed over 3 min. After the trials, the subjects completed a free description questionnaire on bodily sensation.

### 2.5. Statistical Analysis

The results are expressed as mean ± SD. Differences in the data of sensory evaluation between the three beverages were analyzed by the Friedman test. Palm temperatures in the morning and afternoon were analyzed for interaction between beverage and time by two-way analysis of variance (ANOVA). Palm temperatures were compared between the ginger and placebo treatments by paired* t*-tests. Analyses were performed using SPSS Statistics v.25 (IBM, Armonk, NY, USA) and differences were considered significant at* p *< 0.05.

## 3. Results and Discussion

### 3.1. Palatable Ginger Content in the Beverage

**Figure 4:**
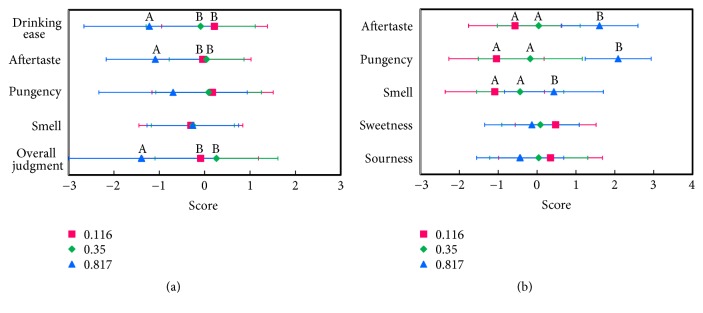
Hedonic (a) and sensory (b) evaluation of ginger-containing beverages. Each value is expressed in terms of hedonic sensation (a) and sensory evaluation (b) among three types of ginger-containing beverages. (a) was a measure of the overall hedonic perception of the drinks containing ginger extract and (b) was a measure of the flavor characteristics of the drinks. The contents of gingerols, including 6-gingerol and 6-shogaol, were 0.116% (■), 0.35% (♦), and 0.817% (▲), and they are shown as mean ± SD (*n *= 23). Test scales were classified as (a) −3 = strongly dislike, −2 = dislike, −1 = slightly dislike, 0 = neither like nor dislike, 1 = somewhat like, 2 = like, and 3 = strongly like and (b) −3 = extremely weak, −2 = weak, −1 = somewhat weak, 0 = moderate, 1 = somewhat strong, 2 = strong, and 3 = extremely strong. Values with different letters indicate a significant difference at* p* < 0.05 (Friedman test).

Three levels of ginger extract content beverages were evaluated by a sensory test. The 0.116% ginger extract beverage obtained the highest score of preference of drinking ease, and this score was significantly higher than that for the 0.817% beverage (*p *= 0.018), but not for the 0.35% beverage (*p *= 0.768, Figure 4(a)). The score of preference for aftertaste was not significantly different between the 0.116% and 0.35% beverages (*p *= 0.338). However, the score of aftertaste for the 0.817% beverage was significantly lower than that for the 0.116% or 0.35% beverages (*p *= 0.001, 0.022, resp.). There was no significant difference in preference for pungency among the three beverages (*p *= 0.063). For overall judgement of palatability, the 0.116% and 0.35% beverages had significantly higher scores than did the 0.817% beverage (*p *= 0.008, 0.027, resp.). The analytical sensory test showed that ginger content level tended to be proportional to the strength of aftertaste and pungency, but there was no significant difference between the 0.116% and 0.35% beverages (Figure 4(b)). Because of no significant difference between the 0.116% and 0.35% beverages in preference for drinking ease and overall judgement, we decided to use a lower content (i.e., 0.116% ginger extract beverage) for human trials on the hyperthermic effect of ginger.

### 3.2. Tympanic Temperature

Tympanic temperature provides a precise indication of deep body temperature [20]. However, with cold exposure treatment, changes in tympanic or rectal temperatures are not significantly different between the* hie-sho* group and non-*hie-sho* group [17, 21]. Therefore, we monitored the subjects' health on the trial days by measuring their tympanic temperature during the acclimatization period before the trial. Among eight subjects, two were excluded from the study because of marked changes in tympanic temperatures between the first and second days. The other six subjects showed stable tympanic temperature within normal limits (changes within 0.5°C), and the results of these subjects were analyzed. The mean tympanic temperatures for these six subjects were 36.5 ± 0.38°C (morning on day 1), 36.6 ± 0.49°C (afternoon on day 1), 36.4 ± 0.42°C (morning on day 2), and 36.4°C ± 0.49°C (afternoon on day 2).

### 3.3. Effect of Ginger Beverage on Skin Surface Temperature

The trial of eight subjects was performed in the morning and afternoon in a crossover manner to account for any effects of circadian variation. However, as described in Section 3.2, palm temperature data for only six subjects were statistically analyzed.

**Figure 5:**
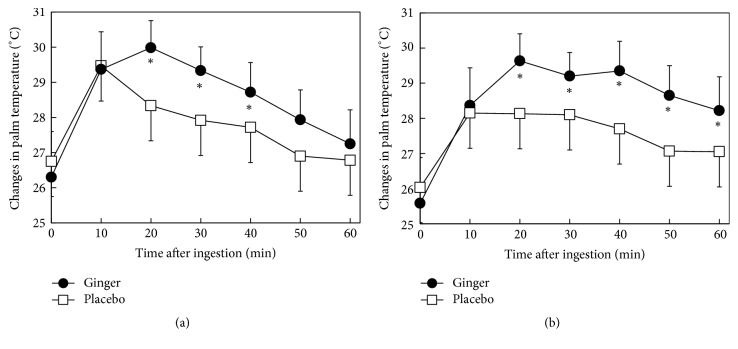
Effects of drinking ginger or placebo beverages on palm temperature in women. Six healthy women with cold sensitivity drank placebo or ginger (677 *μ*g of gingerols, including 6-gingerol and 6-shogaol) beverages in the morning (a) or afternoon (b). Palm temperature was determined by thermographic measurement. Values are shown as mean ± SD for the six women. *∗p *< 0.05 versus the placebo treatment at the same time point (paired* t*-test). There was a significant interaction between beverage and time (*p* = 0.009 in the morning;* p* = 0.024 in the afternoon) by two-way repeated measures ANOVA.

Changes in mean palm temperature after drinking the beverage in the morning trial are shown in Figure 5(a). Palm temperature increased immediately until 10 min after intake in both groups (+3.2°C after ginger and +2.9°C after placebo versus 0 min). However, palm temperature continued to increase until 20 min (up to +3.4°C versus 0 min) after ginger intake, while palm temperature decreased after 10 min (to +2.4°C versus 0 min) after placebo treatment. Palm temperature then decreased in both groups but remained higher in the ginger group compared with the placebo group. ANOVA showed a significant interaction between beverage and time (*p *= 0.009). Paired* t*-tests showed that palm temperatures were significantly higher in the ginger treatment compared with the placebo treatment at 20 (*p *= 0.026), 30 (*p *= 0.025), and 40 min (*p *= 0.038) after intake.

**Figure 6:**
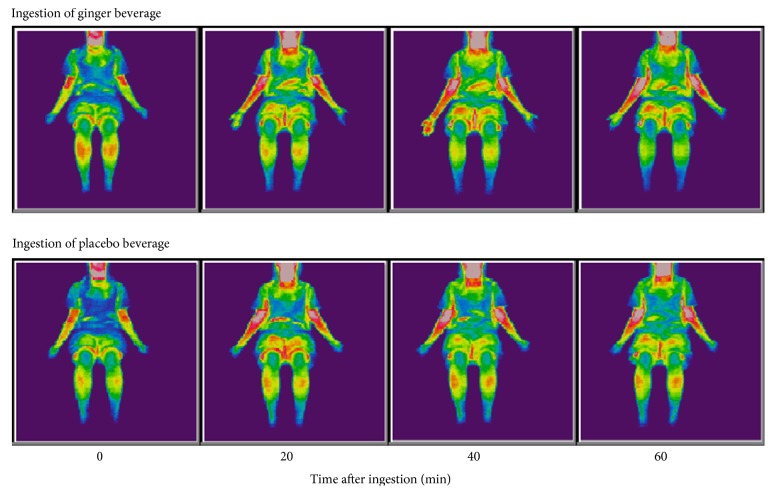
Sample thermograms of body surface temperature changes following ingestion of ginger and placebo beverages. A healthy woman with cold sensitivity (Subject 4) drank a placebo or ginger (677 *μ*g of gingerols, including 6-gingerol and 6-shogaol) beverage in the afternoon. Her body surface temperature was measured by thermography. Her palm temperature was higher at 20, 40, and 60 min after consumption of the ginger beverage compared with the placebo beverage.

Changes in mean palm temperature after drinking the beverage in the afternoon trial are shown in Figure 5(b). A sample thermogram for Subject 4 is shown in Figure 6. Palm temperature increased immediately and continued to increase for 10 min in the ginger and placebo groups (+3.3°C after ginger and +2.3°C after placebo versus 0 min). However, palm temperature after ginger intake continued to increase until 20 min (up to +4.6°C versus 0 min), while palm temperature after placebo intake decreased after 10 min (+2.3°C versus 0 min). Palm temperature after ginger treatment was then maintained until 40 min, while that in the placebo group decreased (+1.9°C versus 0 min). At 60 min, palm temperature in the ginger group had decreased to +3.4°C versus 0 min, while that in the placebo group had decreased to +1.3°C. Repeated measures two-way ANOVA showed a significant interaction between beverage and time (*p *= 0.024). Paired* t*-tests showed that palm temperatures were significantly higher in the ginger treatment compared with the placebo treatment at 20 (*p *= 0.012), 30 (*p *= 0.017), and 40 min (*p *= 0.045) after intake.

Both treatments maintained a higher palm temperature in the afternoon than in the morning. In subjects who drank the ginger beverage, palm temperature after 10 min remained the same after 60 min. Mansour et al. [22] reported that oral intake of dried ginger powder dissolved in hot water enhanced diet-induced thermogenesis and speculated that 6-shogaol was the active component. Because the afternoon trial was performed after taking lunch, the prolonged hyperthermic response may have been due to the additional stimulatory effect of ginger on diet-induced thermogenesis.

Palm temperature increased immediately after intake of either beverage, presumably because of the direct warming effect of the intake of hot liquid (55°C). However, the ginger beverage increased palm temperature more than the placebo beverage in the morning and afternoon trials. Previous trials showed that ginger dissolved in water [15, 23–26] and capsules taken with water [12, 13] also led to elevated body temperature. This evidence suggests that ginger extract has a hyperthermic response, regardless of the beverage temperature.

### 3.4. Subjective Evaluation

Subjective symptoms were assessed by questionnaires after the trials. The answers are shown in Table 1. Five subjects experienced obvious and lasting hyperthermic responses in their peripheral extremities after drinking the ginger beverage. However, some subjects who drank the placebo beverage answered that the hyperthermic response did not last and the warm feeling disappeared quickly, and they did not feel any hyperthermic effect or felt no improvement in cold sensation. One subject noted improved blood flow to her fingertips after consuming the ginger beverage (Table 1). Iwami et al. [27] reported that ginger intake increased energy expenditure and showed a thermogenic effect. However, Fujisawa et al. [15] reported that a lower dosage of ginger did not affect oxygen consumption but expanded the width of peripheral blood vessels and increased blood flow. Fujisawa et al. speculated that even low dosage of gingerols, which does not affect energy metabolism, may have a hyperthermic effect on peripheral skin by increasing the blood circulation.

### 3.5. Dosage of 6-Gingerol and 6-Shogaol (Gingerols) Contents

Gingerols, including 6-gingerol and 6-shogaol, have been reported to be the effective hyperthermic components of ginger [15, 28]. 6-Gingerol converts to 6-shogaol by elimination of a hydroxyl group as a result of treatments, such as heating [29], and this reaction is reversible in aqueous solutions [12, 30, 31]. Therefore, we measured the 6-gingerol and 6-shogaol contents of a beverage containing 0.116% of ginger extract as gingerols. The overall gingerol content was 2.42 ppm, including 2.00 ppm 6-gingerol and 0.42 ppm 6-shogaol. In this study, the gingerol intake was 677 *μ*g per serving (280 mL) for the ginger beverage. No gingerols were detected in the placebo beverage.

We found that a low dosage (677 *μ*g/serving) of gingerols that were consumed in a beverage stimulated a hyperthermic effect at the skin surface of the extremities. Higher doses of gingerols have previously been reported to have hyperthermic effects, including gingerols in water (2.6 mg/serving) [23], in water (6.21 mg/serving) and bread (5.32 mg/serving) [15], in capsules (4.76 mg/serving) [14], and in ginger koji (malted rice) powder (5.40 mg/serving) [12]. Natsuno and Hirayanagi [14] reported that ingestion of a capsule that contained 4.76 mg gingerols per serving resulted in no significant increase in temperature on the thumb but significantly accelerated energy consumption. We speculate that ginger extract in a beverage may increase temperature in the peripheral extremities at a lower dose than in bread or a capsule because of the lack of interference from other components, such as proteins or capsule material.

All of our subjects reported that the ginger extract-containing beverage was easy to drink. In the above-mentioned previous studies, the flavor of the ginger was masked by mixing it in bread [15], capsules [12–14], or tea [16]. No previous taste trials have investigated the taste of ginger samples dissolved in water or hot water [15, 22–26]. However, the beverage in this study was drinkable without a pungent flavor. Commercially available ginger drinks, such as ginger ale, contain low levels of gingerols (data not shown).

## 4. Conclusion

A palatable beverage containing ginger extract has a hyperthermic effect on the peripheral extremities in women suffering from mild cold sensitivity, even at a low dose of gingerols. Therefore, this beverage may help to improve cold sensitivity in people suffering from* hie-sho*.

## Figures and Tables

**Table 1:** Summary of responses to a questionnaire on the hyperthermic effects of a ginger extract-containing beverage (free description) (*n* = 6).

Comment	Responses
Warming continued	5
Warming in fingers and toes	5
Warming from within the body	3
Warming in throat and face	1
Warming in hands and both legs	1
Increased blood flow and pulsation in the fingertips	1

## Data Availability

The data used to support the findings of this study are available from the corresponding author upon request.
